# PADI4 and tumourigenesis

**DOI:** 10.1186/1475-2867-10-7

**Published:** 2010-03-12

**Authors:** Xiaotian Chang, Kehua Fang

**Affiliations:** 1 Laboratory for Bio-Drugs of Ministry of Health, Provincial Laboratory for Modern Medicine and Technology of Shandong, Research Center for Medicinal Biotechnology, Shandong Academy of Medical Sciences, Jingshi Road 18877, Jinan, Shandong, 250062, PR China

## Abstract

PADI4 post-translationally converts peptidylarginine to citrulline, a process called citrullination. Studies have demonstrated the high expression of PADI4 in various malignant tumour tissues. PADI4 is also expressed at high levels in the blood of patients with some malignant tumours. Thus far, citrullination of histone, cytokeratin, antithrombin and fibronectin have been confirmed to be involved in abnormal apoptosis, high coagulation, and disordered cell proliferation and differentiation, all of which are main features of malignant tumours. PADI4 is expressed in CD34+ stem cells in normal tissues, and many more CD34+ cells expressing PADI4 are present in tumour tissues. These findings suggest that PADI4 may play an important role in tumourigenesis.

## Introduction

Peptidylarginine deiminase (PAD) can catalyse peptidyl arginine to citrulline in the presence of Ca^2+ ^ions, a reaction known as citrullination, which leads to the post-translational modification of proteins. Genes encoding PAD family members, including PADI1, PADI2, PADI3, PADI4, and PADI6, cluster at human chromosome position 1p36.13. The high sequence identity of the C-terminal domains of all PADs suggests that their structures are similar [1]. The expression of all of these isoforms has been detected in numerous tissues. PADI1 is found in the skin epidermis [2], PADI2 is present in various tissues including brain and muscle [3,4], PADI3 is localised in hair follicles [5], PADI4 is mainly expressed in granulocytes and monocytes [6], and PADI6 is specially expressed in embryonic stem cells and oocytes [7,8]. Studies have reported that PADs are involved in cell differentiation, apoptosis, nerve growth, embryonic development, and gene regulation [9]. Biochemical and immunohistochemical assays have suggested the involvement of PADI1 in the terminal differentiation of the epidermis [10], PADI2 in the myelination and citrullination of central nerve axons [11,12], and PADI3 in the keratinisation of hair follicles [3,13]. Arginine residues frequently function as ligand recognition sites in proteins. Some enzymes that interact with negatively charged substrates or cofactors have an arginine residue as an anion recognition site [14]. Therefore, it seems possible that PADs and citrullination participate in the regulation of these enzymes. In addition, PAD citrullination was suggested to modify the action of trypsin-like enzymes [15] and trypsin inhibitors [14], to interfere with intermediate filament assembly [16], and to play a role in rapid cellular turnover in tissues with secretory activity [17]. However, the physiological significance of each of the PADs has not been understood in detail.

PADI4 was initially cloned from the HL-60 cell line (human promyelocytic leukaemia cells) [6]. The protein is 663-amino acids long with a molecular weight of 74,095 Da. The PADI4 polypeptide chain has two spherical subunits at the N terminus, one α helix\β fold structure at the C terminus, and five Ca^2+ ^ion-binding sites. The binding of Ca^2+ ^ions to PADI4 induces conformational changes in the peptide chain that result in the formation of active sites, such that the enzyme enters its active state for the catalysis of arginine residues into citrulline residues [1]. PADI4 is predominantly expressed in blood lymphocytes and has been suggested to play a role in inflammation and the immune response [18-21]. Because PADI4 was also detected in granulocytes and macrophages induced by HL60 cells, the enzyme is believed to be involved in the differentiation and apoptosis of these cells [6]. However, little is known about the precise function and target substrates of PADI4 under physiological conditions.

PADs and citrullinated proteins are involved in the pathogenic processes of many human diseases, because citrullination tends to alter the conformation and activity of target proteins, leading to changes in physiological and biochemical activity. Rheumatoid arthritis (RA), an autoimmune disease, is characterised by the presence of many types of autoantibodies against citrullinated proteins in the blood of patients, indicating that citrullination plays an essential role in the autoimmune reaction and the destruction of joint tissues [22,23]. By whole genome SNP scanning, Suzuki et al. demonstrated a strong association of PADI4 with RA [24]. Chang et al. detected an elevated expression of PADI4 in the synovial membrane and synovial fluid of RA patients [19,25]. In addition, Mastronardi et al. detected citrullination of nucleosome histone H3 in the cerebral white matter of multiple sclerosis patients and demonstrated that tumour necrosis factor (TNF)-α induced PADI4 translocation to the nucleus [26]. The citrullination of proteins appears to be abnormally increased in Alzheimer's disease, glaucoma, and neurodegeneration [27-29].

Cancer is one of the leading causes of death in humans. In recent years, our studies and others' found that PADI4 is highly expressed in a variety of malignant tumours and that it participates in the process of tumourigenesis. This review highlights PADI4 expression in tumour tissues as well as the potential role of PADI4 in the pathogenesis of cancer.

## Expression of PADI4 in various malignant tumours

Tumour tissues and synovial tissues from patients with RA have a lot of common characteristics. Both tissues show abnormal cell proliferation, fibrin deposition, coagulation activity, and angiogenesis [30]. Because PADI4 is one of the genes associated with RA, we speculated that PADI4 might also play a role in the tumourigenic process. Using immunohistochemistry, quantitative PCR, and western blot analysis, we detected significant PADI4 expression in tumour cells of various malignancies, including breast carcinomas, lung adenocarcinomas, hepatocellular carcinomas, oesophageal squamous cancers, colorectal adenocarcinomas, renal cancers, ovarian adenocarcinomas, endometrial carcinomas, uterine adenocarcinomas, bladder carcinomas, and chondromas, as well as in other metastatic carcinomas. However, PADI4 was not expressed in benign leiomyomas of the stomach, uterine myomas, endometrial hyperplasias, cervical polyps, teratomas, hydatidiform moles, trophoblastic cell hyperplasias, hyroid adenomas, haemangioma, lymph hyperplasias, schwannomas, neurofibromas, lipomas, or cavernous haemangioma of the liver. Additionally, PADI4 expression was not detected in non-tumour tissues, including cholecystitis, cervicitis, and synovitis of osteoarthritis, except in certain acutely inflamed tissues such as in acute gastritis and acute appendicitis. In addition, western blot analysis detected PADI4 expression in cultured tumour cell lines including A549 (lung adenocarcinoma), SKOV3 (ovarian adenocarcinoma), and U937 (leukaemia) cells. ELISAs detected increased PADI4 levels in the blood of patients with various malignant tumours compared to those in patients with chronic inflammation and benign tumours. PADI4 levels were also significantly associated with CEA levels, a serum marker for tumour diagnosis, in the blood of patients with gastric cancer and prostate cancer [31,32]. Recently, Lv. et al. reported the significantly increased expression of PADI4 in hepatocellular carcinomas compared to that in the surrounding healthy tissues, as determined by western blot analysis [33]. Stacey et al. conducted a genome-wide SNP association study and found that common variants on rs7538876 of 1p36, containing the PAD locus, are significantly associated with coetaneous basal cell carcinoma but not with melanoma or pigmentation traits [34].

Many studies have investigated the tissue distribution of PADI4 in diseased tissues. In the RA synovial membrane, PADI4 was located in T cells, B cells, granulocytes, macrophages, and capillary endothelial cells [19]. PADI4 was also detected in eosinophils and neutrophils of peripheral blood cells [18,19]. In our study, PADI4 was demonstrated in CD34+ cells of the bone marrow and normal tissues by double immunolabelling. Although tumour cells that express PADI4 lacked CD34 signals, the enzyme is located in CD34+ mesenchymal cells in the stromal region of the tumour tissues [31]. These observations and studies by others suggest that PADI4 expression may be limited to haematopoietogenic lineage cells that originally develop from haematopoietic cells (HPCs) in the bone marrow. HPCs marked with CD34 comprise a well-characterised population of multi-potent progenitor cells that are able to self-renew and give rise to terminally differentiated haematopoietic cells, or something more specific. These stem cells can also differentiate and integrate into various tissues such as skeletal muscle, cardiac monocytes, vascular endothelium, liver, and brain [35,36]. Because there are more CD34+ cells expressing PADI4 in tumour tissues than in normal tissues [31], it is postulated that the development of PADI4-expressing tumour cells may be associated with the abnormal proliferation of CD34+ stem cells or their progeny. In fact, many studies have found a strong association between CD34 cells and tumourigenesis in the lung, pancreas, liver, and stomach adenocarcinomas [37-42]. Strobel et al. reported that human bone marrow cells have the ability to stimulate the growth, in a dose-dependent manner, of many types of tumour cells in vitro [43]. Hellwig et al. found that basic fibroblast growth factor (bFGF) and vascular endothelial growth factor (VEGF), both of which are increased with the progression of tumourigenesis, suppress the expression of CD34 in renal carcinoma. Such a down-regulation of CD34 adhesion molecules may allow tumour cells to escape immune surveillance [44]. Their findings may explain the disappearance of CD34 expression in PADI4-expressing tumour cells observed in our study.

## Citrullination of proteins

PADI4 exerts post-translational modifications through the conversion of arginine to citrulline. Therefore, the identification of PADI4 substrates is helpful for understanding the pathogenic mechanisms of the enzyme during tumourigenesis.

P53 inhibits malignant transformation through the transcriptional regulation of its target genes to mediate the cell cycle and apoptosis [45,46]. Under normal circumstances, damaged, aging, or diseased cells increase their expression of p53, activating its downstream target genes and eventually inducing apoptosis to ensure the health of the body [47]. Studies have indicated that p53 regulates the transcription of target genes by post-translational modification of histones via methylation, acetylation, phosphorylation, ubiquitination, and citrullination [48,49]. Using chromosome immunoprecipitation, Yao et al. and Li et al. investigated the promoters of p53 target genes and found that over-expression of PADI4 may prevent arginine methyltransferase from methylating histone H3 by converting arginine residues into citrulline. As a result, the expression of p53 target genes is reduced, resulting in the disruption of cellular apoptosis and the normal cell cycle [50,51]. Hagiwara et al. and Cuthbert et al. also confirmed that the citrullination of histones by PADI4 antagonises arginine methylation [52,53]. Using cultured human leukaemia HL-60 cells and human acute T leukaemia Jurkat cells, Liu et al. found that the over-expression of PADI4 could induce the up-regulation of p53, p21, and Bax expression, resulting in cell cycle arrest and mitochondria-mediated apoptosis [54]. However, Lv et al. recently detected decreased p53 levels in tumour tissues from patients with hepatocellular carcinomas compared to the surrounding healthy tissues. They also found that the expression of p53 was obviously decreased in HeLa cells transfected with a PcDNA3.0-Flag-PADI4 plasmid, suggesting that the enzyme induces tumourigenesis by down-regulating p53 expression [33].

Cytokeratin (CK), the intermediate filament protein, constitutes the cytoskeleton and plays an important role in cellular stress, signal transduction, and apoptosis. CK, as a tumour-specific marker, displays high expression in gastric cancer, pancreatic cancer, breast cancer, urinary tract transitional cell carcinoma, and other tumour epithelial cells [55,56]. Omary et al. have found that the glycosylation, phosphorylation, and other post-translational modifications of CK change its solubility and other physical and chemical properties that lead to cellular apoptosis in a variety of physiological processes [57]. By immunohistochemistry and western blotting, we found that PADI4 and CK have similar distributions in the colon, oesophagus, bladder, lung, rectum, stomach and breast cancer, thyroid tumour, oesophageal squamous cell carcinoma, hepatocellular carcinoma, and other non-adenocarcinomas. Co-immunoprecipitation indicated the citrullination of CK8, 18, and 19 in tumour tissues. Following the citrullination of PADI4, CK is not digested by caspase, thereby disrupting the process of apoptosis [31]. An imbalance of apoptosis is one of the characteristics of malignant tumours.

Antithrombin (AT) is a member of the serine protease inhibitor (serpin) superfamily, and it inhibits plasma thrombin activity to maintain the dynamic equilibrium of the coagulation and anti-coagulation system. Structural changes in AT may lead to abnormal blood clotting. It is reported that approximately 90% of cancer patients are at risk of thrombosis [58]. We incubated recombinant PADI4 with human AT. Western blots and ELISA showed that the citrullination of AT decreased its ability to inhibit thrombin [59]. Recently, Ordóñez A. et al. confirmed by proteomic analysis that the citrullination of antithrombin abolished its activity. They also found that this abolition of activity was accelerated by heparin, which facilitated the citrullination of Arg393 (P1 residue). Their proteomic analyses revealed nine additional citrullines that caused a significant decrease in its electrostatic potential [60]. Using sandwich ELISA, we detected higher levels of PADI4 and citrullinated AT levels in the plasma of patients with malignant tumours, including those with breast carcinomas, hepatocellular carcinomas, lung carcinomas, oesophageal carcinomas, gastric cancer, colon cancer, rectal cancer, pancreatic cancer, ovarian carcinomas, bladder carcinomas, uterine myomas, thyroid carcinomas, and prostate carcinomas. The levels of citrullinated antithrombin were significantly associated with PADI4 levels in blood of patients with hepatocellular carcinomas, lung cancer, ovarian cancer, endometrial carcinomas, and thyroid adenomas. In addition, the levels of citrullinated AT were clearly associated with the CEA levels in the blood of patients with breast carcinomas, bladder cancer, renal cell carcinomas, and pancreatic cancer; with CA199 levels in those with gastric cancer; with CA125 levels in those with ovarian cancer; and with PSA in those with prostate cancer [32]. CEA, CA199, CA125, and PSA are tumour makers and are currently used for tumour diagnosis. These results suggest that PADI4 may be responsible for the high coagulation activity induced by the citrullination of AT in cancer patients. Studies have shown that an increase in thrombin activity can promote fibrin deposition, angiogenesis, and tumour cell invasion and metastasis, ultimately leading to the malignant proliferation of tumour cells [61].

Fibronectin (Fn) is a glycoprotein that is widely present in the extracellular matrix, plasma, and other body fluids. Structurally, it contains multiple functional domains that interact with the extracellular matrix, integrin receptors, and growth factors, which play important roles in cell adhesion, migration, differentiation, and proliferation. A number of studies have demonstrated an important role of Fn in tumourigenesis [62,63]. Our in vitro study showed that rabbit PAD may catalyse citrullination of both the cellular and serum Fn. Following citrullination, the affinity of Fn for VEGF increases, but its binding activity to integrin β 1 decreases, and Fn no longer stimulates the apoptosis of monocytes induced from cultured HL-60 cells [64]. Therefore, it is suggested that Fn may also be citrullinated by PADI4 in tumours, thus leading to tumour cell invasion, proliferation, and malignancies.

## Regulatory mechanism of PADI4

To date, little is known about the mechanisms that regulate PADI4. Dong et al. found that oestrogen regulates the expression of PADI4 through both the classical and non-classical pathways [65]. In the classical way, oestrogen binds to the oestrogen receptor to form a complex and then regulates PADI4 expression via the oestrogen response element on the promoter. In the non-classical pathway, the oestrogen-oestrogen receptor complex acts on the transcription factors AP-1, NF-Y, and SP-1, which bind to the PADI4 promoter to specifically regulate PADI4 expression. On the other hand, Cuthbert et al. found that PADI4 could suppress the expression of oestrogen-regulated genes by antagonizing the methylation of histone H3 on the pS2-promotors of these genes by citrullination [53]. It is possible that oestrogen regulates oestrogen-sensitive genes by a negative feedback effect via the citrullination of PADI4. Recently, Tanikawa et al. reported that p53 transactivated PADI4 through a p53-binding site located in the first intron. Furthermore, they found that the knockdown of PADI4 attenuated p53-mediated growth-inhibitory activity, demonstrating the significance of PADI4-mediated protein citrullination in the p53 signalling pathway [66]. This result seems controversial in light of the findings that PADI4 disrupts the apoptotic process via the citrullination of histone H3 on the promoter of p53-target genes [50,51], but it is consistent with the result that over-expression of PADI4 can induce apoptosis [54].

**Figure 1:**
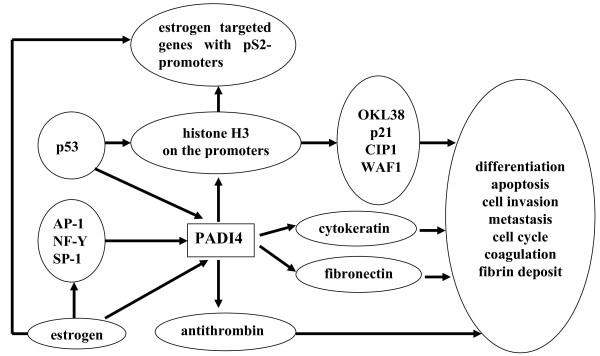
**regulatory mechanism of PADI4 - study summary**.

We review the following regulatory mechanism of PADI4 by summarizing our studies and those of others (Figure 1).

## PADI4, a potential biomarker

To date, a number of biomarkers have been used for tumour diagnosis. However, the specificity and sensitivity are inappropriate for large-scale surveys of tumour prognosis, because these tumour markers can only be expressed in one or several kinds of tumours. PADI4 is highly expressed in a variety of malignant tumours but is either not expressed or is expressed at very low levels in normal tissues and benign tissues. The enzyme can be considered as an immunohistochemical barometer to distinguish tumour cells from non-tumour cells and to define the tissue structure of tumours. Because patients with various malignant tumours display an increased expression of PADI4 in their blood, and because the level of expression declines after tumour excision surgery [32], the enzyme can be used as a serum maker of tumours to compensate for some of the deficiencies in the current methods employed for tumour diagnosis. In addition, the specific nature of PADI4 expression in tumour cells makes PADI4 a potential target for cancer therapy. Hence, PADI4 could have important clinical applications; however, further research is necessary to develop this marker into a useful clinical diagnostic tool.

## Conclusion

In summary, PADI4 demonstrates significant expression in various malignant tissues but maintains a low level of expression in normal and benign tissues. Studies have indicated that PADI4 may be involved in tumourigenesis via the citrullination of histone, cytokeratin, antithrombin, and fibronectin; although some studies suggest that the enzyme can induce apoptosis.

## Competing interests

The authors declare that they have no competing interests.

## Authors' contributions

XC and KF contributed equally to the elaboration of the review. XC is the senior author. All authors read and approved the final manuscript.
